# Multimorbidity as a Predictor as Mortality in Companion Dogs

**DOI:** 10.1093/geroni/igaf122.966

**Published:** 2025-12-31

**Authors:** Jessica Hoffman

**Affiliations:** Augusta University, Augusta, Georgia, United States

## Abstract

Multimorbidity, the co-occurrence of two or more diseases, increases with age and is a predictor of mortality in humans; however, modeling multimorbidity in laboratory models is difficult if not impossible. Here, we utilize the companion dog as a translational model of multimorbidity, tracking over 50,000 dogs in the Dog Aging Project (DAP) for up to five years. Similar to humans, we found that the accumulation of multiple conditions is associated with an increased risk of mortality in dogs. In addition, dogs with a diagnosis of osteoarthritis or other degenerative joint diseases at baseline entry into the DAP exhibited significantly higher diagnosis rates and a higher likelihood of developing multimorbidities. Interestingly, we then determined that specific co-morbid disease combinations were more likely to be associated with mortality than individual disease effects. For example, dogs with several periodontal diseases and heart murmurs were at an increased risk of death compared to dogs with either condition on its own. Overall, our study is the first to investigate the effects of multimorbidity on mortality in companion dogs, and future studies will attempt to determine if circulating biomarkers can predict future multimorbidity.

